# Long-term neurocognitive and educational outcomes of neonatal insults in Kilifi, Kenya

**DOI:** 10.1186/s12888-020-02939-9

**Published:** 2020-12-02

**Authors:** Dorcas N. Magai, Hans M. Koot, Paul Mwangi, Esther Chongwo, Charles R. Newton, Amina Abubakar

**Affiliations:** 1 Centre for Geographic Medicine Research Coast, Kenya Medical Research Institute, P.O Box 230, Kilifi, Kenya; 2 Department of Clinical, Neuro- and Developmental Psychology, Amsterdam Public Health Research Institute, Vrije Universiteit Amsterdam, Van der Boechorststraat 1, 1081 BT Amsterdam, The Netherlands; 3 Department of Public Health, Pwani University, P.O. Box 195-80108, Kilifi, Kenya; 4 Department of Psychiatry, University of Oxford, Oxford, OX3 7JX UK; 5 Institute for Human Development, Aga Khan University, P.O. Box 30270-00100, Nairobi, Kenya

**Keywords:** Disability, Neurocognitive impairment, Children, Neonatal jaundice, Hypoxic-ischemic encephalopathy

## Abstract

**Background:**

There is little data on the long-term neurocognitive and educational outcomes among school-aged survivors of neonatal jaundice (NNJ) and hypoxic-ischemic encephalopathy (HIE) in Africa.

This study investigates the long-term neurocognitive and educational outcomes and the correlates of these outcomes in school-aged survivors of NNJ or HIE in Kilifi, Kenya.

**Methods:**

We conducted a cross-sectional study on neurocognitive and educational outcomes among school-aged survivors (6–12 years) of NNJ (*n* = 134) and HIE (*n* = 107) and compared them to a community comparison group (*n* = 134). We assessed nonverbal intelligence, planning, working memory, attention, syntax, pragmatics, word-finding, memory, perceptual-motor, mathematical, and reading abilities. We also collected information on medical history, caregivers’ mental health, and family environment.

**Results:**

The survivors of NNJ had lower mean total scores in word-finding [F (1, 250) = 3.89, *p* = 0.050] and memory [F (1, 248) = 6.74, *p* = 0.010] than the comparison group. The survivors of HIE had lower mean scores in pragmatics [F (1, 230) = 6.61, *p* = 0.011] and higher scores higher scores in non-verbal reasoning [F (1, 225) =4.10, *p* = 0.044] than the comparison group. Stunted growth was associated with almost all the outcomes in HIE.

**Conclusion:**

Survivors of NNJ and HIE present with impairment in the multiple domains, which need to be taken into consideration in the planning of educational and rehabilitative services.

## Background

Neonatal jaundice (NNJ) and hypoxic-ischemic encephalopathy (HIE) are common conditions especially in Africa [1, 2] and are significant causes of childhood neurodevelopmental and neurological impairment, and neonatal mortality [3]. Most studies have focused on the neurodevelopmental outcomes of survivors of NNJ [4–6] and HIE [7, 8] at earlier ages and only a few studies have explored the long-term consequences for survivors of these conditions at school age [9–18].

The few available studies present inconsistent findings on the long-term neurocognitive and educational outcomes in school-aged survivors of NNJ or HIE. Some studies have reported low risk of physical [18]; neurocognitive and neurological [12, 19, 20]; language and speech [21, 22] and hearing impairment [22] in survivors of NNJ. Similarly, several studies have indicated low risk of poor school performance [16, 17], neuropsychological [17], cognitive impairment or physical disability [9, 15, 23] in survivors of moderate HIE. In contrast, cognitive impairment and poor academic achievement [11] and an increased risk for neurodevelopmental disorders and speech and language deficits later in life have been reported in other studies of survivors of NNJ [19, 24] and survivors of mild and moderate [9, 13, 14], and severe [15–17] HIE.

Although these studies provide information on the long-term neurocognitive and educational outcomes of school-aged survivors of NNJ or HIE, all the studies are from high-income countries, and little is known about the outcomes of school-aged survivors of NNJ or HIE in low-income settings such as those in sub-Saharan Africa where the incidence of NNJ and HIE is high. Moreover, the long-term neurocognitive and educational outcomes can only be understood in the context of dynamic models of human development such as the bioecological model that proposes an interaction of both biological and environmental factors that influence child’s development [25]. Yet little has been documented about the correlates of neurocognitive and educational outcomes in survivors of NNJ and HIE. This study investigates the long-term neurocognitive and educational outcomes and the correlates of these outcomes in school-aged survivors of NNJ or HIE in Kenya.

## Methods

### Study design

This is a cross-sectional study of children aged six to 12 years admitted with NNJ or HIE at Kilifi County Hospital (KCH).

### Setting

The study was conducted at the Centre for Geographic Medicine Research - Coast (CGMR-C) situated at the Kenyan coast. All study procedures and assessments were conducted at the CGMR-C Neuroscience unit. We utilised the Kilifi Health Demographic Surveillance system (KHDSS) to recruit children who were admitted with severe NNJ and HIE. The KHDSS covers an area of 891 KM^2^ with an approximate population of 265,000 residents [26]. The system is updated quarterly and captures information about the location of the households, births and deaths, and residents’ immigration and outmigration information, etc. [26]. The residents are also matched with the patients register at the KCH at various hospital entry points, and the master KHDSS database is updated weekly.

### Participants

Children who took part in this study were born between 2005 to 2012 and were admitted to KCH in their neonatal period with a diagnosis of either NNJ or HIE. The diagnosis of NNJ was based on clinical laboratory measurement of total serum bilirubin (TSB) as well as medical history and examination at admission. NNJ was defined as a TSB level of > 85 μmols/l recorded in the clinical notes [27]. Our inclusion of children with TSB > 85 μ/mols/l is based on two facts: First, this is the level at which jaundice is reliably detected in the neonate. It is the definition used by the American Academy of Pediatrics for hyperbilirubinemia, and other authors [27–30]. Second, there are considerable difficulties in establishing gestational age [31, 32] and time of birth of neonates admitted to hospitals serving rural areas in sub-Saharan Africa, where most deliveries occur at home, and neonates are admitted after the onset of NNJ in the community. Moreover, there is considerable debate about the criteria for a safe level of bilirubin in sick neonates [33–35].

HIE diagnosis was based on the clinical diagnosis recorded by a clinician. HIE diagnosis was given if a child; had convulsions, was unable to breastfeed, had apnoea, and or poor motor tone [36]. The participants in the comparison group were identified through the KHDSS and were included in the study if they did not have any history of hospital admission.

### Measurement

#### Child-level data

##### Neurocognitive measures

All the participants were assessed using a battery of tests which took approximately two and a half hours to complete. The battery was completed by research assistants trained in neurocognitive assessment. The battery comprised of the following tests:

Nonverbal intelligence was assessed with the *Raven’s coloured progressive matrices (RCPM)* [37]. The RCPM has been adapted and used in Kilifi and has sound internal consistency (ICC = 0.81) [38]. Executive functioning was assessed using the *Tower of London* test, which measures a child’s planning and problem-solving ability [39]. Working memory was assessed with the *Self-order Pointing Test* [40]. Visual attention was assessed with the *People Search Test*. The children are presented with a sheet of silhouette drawings organized in rows, and the child’s task is to draw a line through a target picture while avoiding other pictures [38]. The test has been adapted and used in Kilifi [38].

A child’s use of grammar and sentence structure; functional language use; and size and breadth of vocabulary were assessed with *syntax, pragmatics*, and *word-finding tests*, respectively. The test battery was developed and previously used in Kilifi [41]. Memory was assessed with the *Kilifi Creek Behavioral Memory Test for children (KCBMT)* [42], while perceptual-motor was assessed with the *Purdue Pegboard Test*. Purdue Pegboard test was validated for the population in Kilifi, Kenya, and has been used in studies of neurocognition among children with malaria [38].

##### Educational outcomes

Mathematical and reading skills were assessed with the *Early Grade Mathematical Assessment (EGMA)* and *Early Grade Reading Assessment (EGRA)* [43], respectively. Both EGMA and EGRA have been adapted for use in the Kenyan population [43].

##### Anthropometry

For each child, anthropometric measurements were taken. The calculations of height-for-age (HAZ) and weight-for-age (WAZ) were carried out using the World Health Organization Anthro plus for personal computers version 3.2.2 [44].

##### Medical history and neurological examination

A trained clinician conducted a physical examination to determine the motor and sensory neuron responses of the children using a detailed neurological proforma adapted for this study, from a proforma that has been extensively used within the study setting [4]. The neurological examination items were coded into a variable indicating whether a child had neurological problems or not. The clinician also conducted a medical history to document other potential biomedical risk factors such as abnormal pregnancy (defined as post-dated pregnancy, bleeding during pregnancy, pre-eclampsia, or any other health problems during pregnancy), place of birth (home versus hospital), abnormal delivery (defined as postpartum haemorrhage, emergency caesarean section, prolonged labour, obstructed labour, and maternal and fetal distress), delayed crying at birth, breathing problems at birth, hospital admission, presence of febrile seizures, and presence of any other medical problem after discharge from hospital.

#### Demographic information

Caregivers’ demographic variables that were assessed include sex, age, level of education, marital status, and religion. We also captured information about the child’s sex, age, and number of years of schooling.

#### Caregiver-level data

##### Mental health

Caregivers’ mental health in the past 2 weeks was assessed with the *Patient Health Questionnaire (PHQ-9)* [45]. The participant responds to questions ranging from 0 to 3, depending on how well the statement best describes their situation. The PHQ-9 had excellent internal consistencies in this study (Cronbach’s alpha =0.82).

##### Household level data

The caregivers’ perception of their family life was assessed with the *Family Environment Questionnaire (FEQ)* [46]. The scale has items that assess different components that measure the family environment. The items were summed up to obtain a total score. The FEQ had a relatively low internal consistency in this study (Cronbach’s alpha = 0.50).

The family socioeconomic activities were captured using the *Kilifi Asset Index* [47]. The tool has items that accounts for different assets owned by the family, including electronic devices, livestock, house and land ownership. The participant is expected to indicate how many assets they own. A total score of assets owned was then computed.

### Study size

To investigate the neurodevelopmental outcomes, the sample size calculation was based on results from three previous studies [3, 14]. Based on effect sizes given in these studies, we computed the sample sizes needed in each group. Using G-power 3.1 software calculations, at least 47 participants in the HIE group and 64 participants in the NNJ group are required to give a power of 95% (alpha = 0.05) to detect significant differences between these groups and comparison group. The number of participants in the comparison group was calculated using frequency matching, where 20 participants were required in each age band. Therefore, 140 participants in the comparison group were needed for the seven age bands 6 through 12 years.

### Statistical methods

Student t-test and Chi-square tests were used to compare the differences in participants’ characteristics between the survivors and the comparison group for continuous variables and categorical variables, respectively. We conducted an analysis of variance (ANOVA) or Chi-square test to compare differences among the three groups. We used analysis of covariance (ANCOVA) to examine group differences on the neurocognitive measures adjusting for sociodemographic factors (age, sex, anthropometry, religion, education level, marital status, family socioeconomic status, place of birth, preterm birth, and obstetric complications). The anthropometric variables WAZ and HAZ were standardized using WHO Anthro plus [48]. An abnormal nutritional status (stunted growth or underweight) was considered if the z-scores obtained from WHO Anthro plus were below − 2 standard deviation (SD)*.* We conducted univariate regression analysis to identify factors classified as child factors (stunted growth), parental factors (family asset, education level, marital status, family environment, mental health), obstetric factors (abnormal pregnancy, place of birth, abnormal delivery, delayed crying, feeding problems, hospital admission, febrile seizures, and medical problems (other insults and infections) that are associated with the neurocognitive outcomes. The factors that yielded an association with the *p*-value level ≤ 0.25 were entered in the multivariate regression analysis to investigate correlates of neurocognitive and education outcomes in NNJ and HIE [49]. We did a stepwise regression analysis with four models adjusting for age, sex and years of education.

## Results

**Fig. 1:**
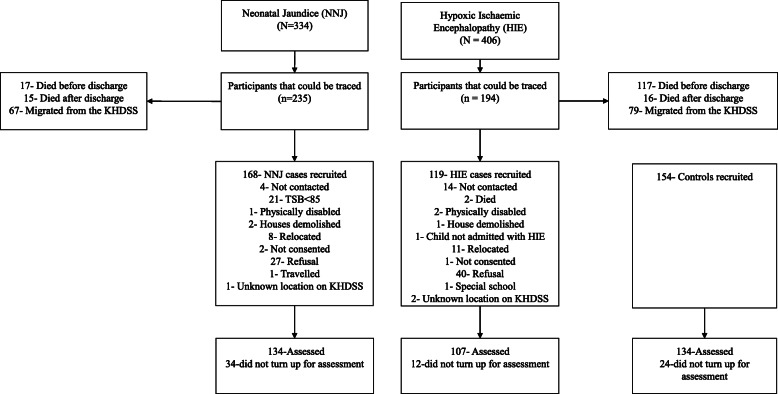
Flow Chart of Identification, Recruitment, and Assessments of Survivors of Neonatal Jaundice and Hypoxic Ischaemic Encephalopathy

This study reports findings from 375 participants; 134 who survived NNJ, 107 who survived HIE and 134 participants in the comparison group. Figure 1 indicates the recruitment process of the participants included in this study. Three participants had incomplete demographic information, but they were retained in the analysis. Most of the participants (57.3%) were males. There were no differences in sociodemographic characteristics among the three groups (*p* > 0.05) except for the significant differences in age, preterm birth, and obstetric complications (Table 1).

**Table 1:** Characteristics of Participants

	*N* = 375	NNJ n (%) = 134 (35.7)	HIE n (%) = 107 (28.5)	Comparison group n (%) =134 (35.7)	NNJ versus Comparison group *p*	HIE versus Comparison group *p*	Overall Comparison Statistics *p*
Sociodemographic characteristics							
Age (years; median [IQR])	9 [7–11]	10 [8–12]	9 [7–11]	9 [7–11]	0.011*	0.352*	0.038***
Sex n (%)							
Female	157 (41.9)	48 (35.8)	44 (41.1)	65 (48.5)	0.051**	0.252**	0.143**
Male	215 (57.3)	83 (61.9)	63 (58.9)	69 (51.5)			
Years of schooling	2.01 (1.7)	2.3 (1.8)	2.0 (1.7)	1.8 (1.4)	0.008*	0.033*	0.052***
Anthropometric data Mean (SD)							
Mid upper arm circumference (cm)	19.14 (7.83)	19.1 (7.8)	19.6 (8.6)	18.8 (7.3)	0.690*	0.424*	0.723***
WAZ	−0.99 (2.08)	−0.76 (2.83)	1–0.10 (2.2)	−1.2 (1.07)	0.240*	0.383*	0.448***
HAZ	−1.19 (1.25)	−1.13 (1.42)	− 1.3 (1.23)	−1.16 (1.10)	0.878*	0.306*	0.498***
Nutritional status n (%)							
Normal	288 (76.8)	103 (76.9)	81 (75.7)	104 (77.6)	0.880**	0.727**	0.941**
Stunted	87 (23.2)	31 (23.1)	26 (24.3)	30 (22.4)			
Parent religion n (%)							
Christianity	260 (69.3)	97 (72.4)	80 (74.8)	83 (61.9)	0.338**	0.118**	0.199**
Islam	34 (9.1)	15 (11.2)	5 (4.7)	14 (10.4)			
Traditional	72 (19.2)	22 (16.4)	20 (18.7)	30 (22.4)			
Parent education level n (%)							
None	131 (34.9)	51 (38.1)	36 (33.6)	44 (32.8)	0.630**	0.933**	0.930**
Primary	197 (52.5)	67 (50.0)	58 (54.2)	72 (53.7)			
Secondary	25 (6.7)	10 (7.5)	7 (6.5)	8 (6.0)			
College/University	13 (3.5)	6 (4.5)	4 (3.7)	3 (2.2)			
Parent occupation n (%)							
Farmer	147 (39.2)	45 (33.6)	48 (44.9)	54 (40.3)	0.450**	0.486**	0.332**
Trader/Business	129 (34.4)	52 (38.8)	29 (27.1)	48 (35.8)			
Casual laborer	55 (14.7)	24 (17.9)	16 (15.0)	15 (11.2)			
Fisher man	1 (0.3)	6 (4.5)	1 (0.9)	0			
Professional	20 (5.3)	7 (5.2)	8 (7.5)	6 (4.5)			
Other	14 (3.7)	0	3 (2.8)	4 (3.0)			
Marital status n (%)							
Not married	60 (16.0)	17 (12.7)	18 (16.8)	25 (18.7)	0.179**	0.712**	0.396**
Married	315 (84.0)	117 (87.3)	89 (83.2)	109 (81.3)			
Family Asset							
Mean (SD)	1.0 (1.4)	1.7 (1.4)	1.8 (1.5)	1.7 (1.4)	0.933*	0.662*	0.895***
median [IQR])	1 (2–2)	1 (1–2)	1 (1–2)	1 (1–2)			
Preterm birth n (%)	13 (3.5)	8 (6)	5 (4.7)	0 (0)	0.004**	0.011**	0.019**
Obstetric complications n (%)							
Abnormal pregnancy	98 (26.9)	37 (27.6)	38 (35.5)	23 (17.2)	0.080**	0.003**	0.007**
Delivery problems	108 (28.8)	73 (68.2)	28 (20.9)	7 (5.2)	0.000**	0.000**	0.001*

Note. WAZ- weight-for-age; HAZ- height-for-age; IQR-Interquartile Range; n-number of participants; * Student t-test; ** Chi-square test; *** Analysis of Variance

### Neurocognitive and educational outcomes in survivors of neonatal jaundice versus the comparison group

The survivors of NNJ had significantly lower mean total scores in word-finding [F (1, 250) = 3.89, *p* = 0.050] and memory [F (1, 248) = 6.74, *p* = 0.010] than the comparison group (Table 2).

**Table 2:** Neurocognitive Functioning and Educational Outcomes in Survivors of Neonatal Jaundice and the Comparison Group

	NNJ (*N* = 134)		Comparison Group (*N* = 134)		Group Differences			
Neurocognitive Outcome	Unadjusted Mean (SD)	Adjusted Mean (SE)	Unadjusted Mean (SD)	Adjusted Mean (SE)	*F*	*df*	*p*-value	Partial Eta Squared
Nonverbal intelligence	11.77 (5.84)	11.06 (0.42)	9.45 (4.38)	10.13 (0.41)	2.29	1244	0.131	0.01
Planning	1.40 (0.99)	1.44 (0.09)	1.61 (1.00)	1.58 (0.09)	0.80	1.243	0.371	0.00
Working memory	94.53 (10.81)	93.90 (0.86)	92.10 (8.85)	92.70 (0.84)	0.92	1245	0.338	0.00
Visual attention	7.48 (4.46)	7.03 (0.30)	6.03 (2.93)	6.46 (0.30)	1.69	1231	0.195	0.01
Syntax	26.11 (8.28)	25.28 (0.56)	24.89 (6.51)	25.71 (0.56)	0.27	1251	0.607	0.00
Pragmatics	86.68 (13.19)	86.79 (0.86)	88.15 (5.16)	88.05 (0.87)	0.92	1253	0.340	0.00
Word finding	37.05 (7.43)	36.89 (0.47)	38.10 (2.45)	38.42 (0.47)	3.89	1250	**0.050**	0.02
Memory	69.98 (23.44)	66.78 (1.56)	69.52 (17.12)	72.71 (1.56)	6.74	1248	**0.010**	0.03
Perceptual-motor	9.87 (2.02)	9.61 (0.14)	9.27 (1.98)	9.53 (0.14)	0.13	1242	0.722	0.00
Educational Outcomes								
Mathematical skills	36.01 (16.87)	33.43 (1.06)	32.33 (16.94)	34.89 (1.05)	1.21	1247	0.273	0.01
Reading skills	86.97 (47.84)	80.90 (2.98)	83.08 (46.32)	89.19 (2.98)	2.95	1249	0.087	0.01

Note: All outcomes were adjusted for age, sex, years of schooling, middle-upper-arm circumference (muac); nutrition status; religion; family asset; parental education and marital status, preterm birth, abnormal pregnancy, and abnormal delivery

### Covariates of neurocognitive and educational outcomes in neonatal jaundice

In the multivariate analysis medium to large portions (18–61%) of the variance in all the neurocognitive and educational outcomes in NNJ were jointly associated with the risk domains except for planning and problem solving (*p* = 0.192). Stunted growth was associated with reduced syntax scores (β = − 0.20, *p* = 0.012). Abnormal pregnancy was associated with reduced pragmatics scores (β = − 0.22, *p* = 0.007); and febrile seizures was associated with reduced perceptual-motor scores (Table 3).

**Table 3:** Correlates of Neurocognitive and Educational Outcomes in Survivors of Neonatal Jaundice

	Nonverbal intelligence	Planning	Working memory	Visual attention	Syntax	Pragmatics	Word finding	Memory	Perceptual motor	Mathematical skills	Reading skills
Risk factors	β (95% CI)	β (95% CI)	β (95% CI)	β (95% CI)	β (95% CI)	β (95% CI)	β (95% CI)	β (95% CI)	β (95% CI)	β (95% CI)	β (95% CI)
Step 1 Child factors											
Child’s age in years	−0.05(− 0.77–0.47)	–	0.23(− 0.11–2.46)	0.29*(0.13–1.09)	**0.18(− 0.14–1.59)**	–	− 0.10 (−1.25–0.53)	0.20*(0.03–4.51)	0.37**(0.17–0.56)	0.24**(0.53–3.45)	− 0.01(−4.58–3.91)
Child’s female sex	–	–	–	–	0.09(− 0.83–4.02)	0.08(−2.21–6.75)	0.13(− 0.49–4.64)	–	–	0.07(− 2.00–6.59)	0.13*(0.07–25.80)
Stunted growth	–	–	–	–	− 0.20* (−6,95- -0.90)	− 0.13 (− 9.16–1.13)	−0.10 (− 4.96–1.30)	–	–	–	–
Years of education	0.58**(1.20–2.62)	–	0.10(− 0.89–2.13)	0.27*(− 0.11–1.23)	0.35**(0.71–2.67)	0.18*(0.14–2.56)	0.36**(0.59–2.64)	0.39**(2.54–7.78)	0.32**(− 0.16–0.60)	0.50**(3.10–6.45)	0.71**(14.21–24.12)
Step 2 Parental factors											
Family asset	–	–	–	0.14 (− 0.18–0.99)	–	–	–	–	**–**	**–**	0.07 (− 2.04–7.53)
Education level	–	–	–	–	–	–	–	–	–	–	–
Marital status	0.14 (−0.09–5.15)	0.11 (− 0.21–0.84)		0.11 (− 0.60–3.50)	–	–	–		–	–	–
Family environment	0.05 (− 0.21–0.44)	–	–	–	0.06 (− 0.24–0.62)	–	–	–	–	–	–
Mental health	–	–	–	–	–	–	–	0.12 (− 0.08–1.08)	–	–	–
Step 3 Obstetric factors											
Abnormal pregnancy	**–**	0.14 (− 0.11–0.69)	–	–	–	− 0.22** (− 11.31- -1.86)	–	–	–	–	–
Place of birth	0.05 (− 1.21–2.45)	−0.05 (− 0.45–0.27)	–	0.05 (− 1.05–1.97)	−0.05 (− 1.65–3.47)	–	–	–	0.05 (− 0.39–8.00)	**−**0.03 (−3.24–5.60)	0.06 (− 7.39–19.76)
Abnormal delivery	–	–	− 0.10 (− 7.24–1.97)	−0.11 (− 1.05–1.97)	−0.03 (− 3.52–2.39)	–	− 0.10 (− 4.86–1.17)	–	−0.05 (− 0.94–0.49)	–	− 0.13 (− 30.84–0.86)
Delayed crying	–	−0.11 (− 0.98–0.24)	0.08 (− 0.08–0.89)	–	–	–	0.01 (− 4.54–4.10)	–	−0.02 (− 1.15–0.92)	–	–
Feeding problems	–	–	–	− 0.07 (− 0.90–2.26)	–	–	–	–	0.06 (− 0.38–0.93)	–	−0.03 (− 17.55–10.76)
Hospital admission		–	–	–	–	–	–	–	–	–	–
Febrile seizures	–	–	–	–	–	−0.08 (−10.64–3.88)	–	–	− 0.22** (− 2.28- -0.48)	–	–
Medical problems		0.12 (− 0.19–1.00)	−0.14 (− 11.64–1.16)	–	0.07 (− 2.10–6.09)-	–	–	–	–	–	–
Neurological problems	–	–	− 0.14 (− 7.81–1.02)	–	− 0.26 (− 7.93- -2.29)	−0.28** (− 13.81- -3.68)	−0.36** (− 9.48- -3.59)	−0.25 (− 21.34- -6.34)	−0.04 (− 0.85–0.48)	−0.16 (− 11.18 - -1.21)	−0.19** (− 35.31- -6.60)
Hyperbilirubinemia	–	–	–	–	–	–	**–**	–	–	–	0.04 (− 8.67–15.94)
R^2^ (*p*)	0.33 (0.000)	0.06 (0.192)	0.18 (0.002)	0.37 (0.000)	0.46 (0.000)	0.21 (0.000)	0.32 (0.000)	0.42 (0.000)	0.53 (0.000)	0.57 (0.000)	0.61 (0.000)

Note: * *p* < 0.05; ** *p* < 0.001; Dash line (−)- the variable was not carried forward to the multivariate analysis

### Neurocognitive and educational outcomes in survivors of hypoxic-ischemic encephalopathy versus the comparison group

The survivors of HIE had significantly lower mean scores in pragmatics scores [F (1, 230) = 6.61, *p* = 0.011] than the comparison group. However, the HIE group had significantly higher mean non-verbal reasoning scores [F (1, 225) = 4.10, *p* = 0.044] than the comparison group (Table 4).

**Table 4:** Neurocognitive and Educational Outcomes in Survivors of Hypoxic-Ischemic Encephalopathy and the Comparison Group

	HIE (*N* = 107)		Comparison Group (*N* = 134)		Group Differences			
Neurocognitive Outcome	Unadjusted Mean (SD)	Adjusted Mean (SE)	Unadjusted Mean (SD)	Adjusted Mean (SE)	*F*	*df*	*p*-value	Partial Eta Squared
Nonverbal intelligence	11.60 (5.73)	11.22 (0.48)	9.45 (4.38)	9.76 (0.42)	4.10	1225	**0.044**	0.02
Planning	1.33 (1.00)	1.38 (0.12)	1.61 (1.00)	1.57 (0.11)	1.01	1222	0.316	0.01
Working memory	92.60 (9.13)	92.04 (0.95)	92.10 (8.85)	92.53 (0.81)	0.12	1222	0.729	0.00
Visual attention	5.79 (2.39)	5.66 (0.26)	6.03 (2.93)	6.13 (0.22)	1.44	1210	0.232	0.01
Syntax	23.86 (8.00)	23.54 (0.59)	24.89 (6.51)	25.19 (0.52)	0.12	1225	0.729	0.00
Pragmatics	86.01 (11.60)	84.99 (1.03)	88.15 (5.16)	88.98 (0.90)	6.61	1230	**0.011**	0.03
Word finding	36.80 (6.99)	37.02 (0.56)	38.10 (2.45)	37.93 (0.48)	1.17	1224	0.281	0.01
Memory	69.99 (28.69)	70.91 (2.36)	69.52 (17.12)	68.77 (2.06)	0.36	1225	0.551	0.00
Perceptual motor	9.09 (2.30)	8.95 (0.20)	9.27 (1.98)	9.38 (0.17)	2.08	1217	0.151	0.01
Mathematical skills	33.88 (16.64)	33.15 (1.35)	32.33 (16.94)	32.93 (1.18)	0.01	1225	0.915	0.00
Reading skills	83.56 (46.06)	82.05 (3.86)	83.08 (46.32)	84.32 (3.38)	0.15	1225	0.699	0.00

Note: All outcomes were adjusted for age, sex, years of schooling, middle-upper-arm circumference (muac); nutrition status; religion; family asset; parental education and marital status, preterm birth, abnormal pregnancy, and abnormal delivery

### Correlates of neurocognitive and educational outcomes in hypoxic-ischemic encephalopathy

Medium to large portions (17–61%) of the variance in all the neurocognitive and educational outcomes in HIE were accounted for by the correlates in the three risk domains. (Table 5).

**Table 5:** Correlates of Neurocognitive and Educational Outcomes in Survivors of Hypoxic-Ischemic Encephalopathy

	Nonverbal intelligence	Planning and problem	Working memory	Visual attention	Syntax	Pragmatics	Word finding	Memory	Perceptual motor	Mathematical skills	Reading skills
Risk factors	β (95% CI)	β (95% CI)	β (95% CI)	β (95% CI)	β (95% CI)	β (95% CI)	β (95% CI)	β (95% CI)	β (95% CI)	β (95% CI)	β (95% CI)
Step 1 Child factors											
Child’s age in years	0.14(− 0.34–0.91)	0.17(− 0.01–0.18)	0.38**(0.61–2.70)	0.28*(0.07–0.58)	0.25*(− 0.13–1.74)	–	0.18 (− 0.21–1.40)	0.13(− 1.72–5.53)	0.37**(0.16–0.65)	0.39**(1.73–4.54)	0.35**(4.03–11.61)
Child’s female sex	–	–	–	–	–	–	–	–	–	–	–
Stunted growth	− 0.16* (−4.04- -0.21)	–	− 0.20* (−8.02- -0.49)	− 0.20* (− 2.01 - -0.16)	−0.29** (−8.17- -2.43)	− 0.15 (−9.19–1.18)	−0.30** (−7.63- -1.88)	−0.31** (− 33.70- -8.39)	−0.08 (− 1.31–0.49)	−0.24** (− 14.52- -4.22)	−0.24** (− 39.83- -11.86)
Years of Education	0.56** (1.19–2.45)	–	− 0.08 (− 0.87–1.67)	0.35** (− 0.18–0.79)	0.35** (0.66–2.57)	0.17 (0.20–2.46)	0.18 (−0.25–1.68)	0.28* (0.54–9.11)	0.25*(0.04–0.63)	0.39**(2.00–5.41)	0.42**(6.37–15.71)
Step 2 Parental factors											
Education level	–	0.17 (− 0.05–0.78)	–	–	–	–	− 0.08 (− 3.76–1.55)	–	–	–	–
Marital status	–	–	–	–	− 0.12 (− 5.82–0.92)	–	− 0.09 (− 5.08–1.81)	–	–	–	–
Family environment	−0.03 (− 0.25–0.38)	–	− 0.06 (− 0.84–0.40)	–	–	–	–	–	–	−0.02 (− 0.99–0.71 2.16)	−0.01 (− 2.49–2.09)
Mental health	–	–	–	–	–	− 0.12 (− 0.82–0.21)	− 0.19* (− 0.60- -0.01)	–	0.02 (− 0.08–0.10)	–	–
Step 3 Obstetric factors											
Abnormal pregnancy	–	–	–	–	–	–	–	–	0.06 (− 0.57–1.10)	–	0.00 (− 12.70–12.65)
Place of birth	–	–	–	–	–	–	–	0.06 (− 10.61–20.34)	–	–	–
Abnormal delivery	–	–	–	–	− 0.16* (− 5.47- -0.12)	–	–	− 0.05 (− 16.22–0.942)	–	–	–
Delayed crying	–	–	0.07 (−2.31–5.17)	–	–	–	0.06 (− 2.07–3.86)	0.19 (− 0.34–25.69)	–	–	–
Breast Feeding problems	–	–	–	− 0.13 (− 1.46–0.19)	–	–	–	–	–	–	–
Febrile seizures	–	–	–	–	− 0.16 (− 6.20- -0.18)	− 0.13 (− 9.15–1.94)	–	− 0.17 (− 26.31–1.47)	–	–	–
Hospital admission	−0.09 (− 5.01–1.12)	–	–	–	–	–	− 0.01 (− 4.99–4.42)	–	−0.19* (− 3.08- -0.22)	–	− 0.05 (− 30.23–14.31)
Medical problems	–	–	–	–	–	–	–	–	–	–	–
Neurological problems	−0.03 (− 2.28–1.44)	–	− 0.14 (− 6.56–0.96)	–	− 0.14 (− 5.33–0.44)	− 0.21* (− 10.48- -0.30)	−0.20* (− 5.98- -0.10)	−0.00 (− 13.30–12.93)	−0.01 (− 0.84–0.90)	−0.01 (− 5.43–4.55)	−0.09 (− 1.38–0.17)
R^2^ (P)	0.50 (0.000)	0.25 (0.036)	0.30 (0.000)	0.39 (0.000)	0.48 (0.000)	0.17 (0.005)	0.34 (0.000)	0.34 (0.000)	0.40 (0.00)	0.58 (0.000)	0.61 (0.000)

Note: * *p* < 0.05; ** *p* < 0.001; Dash line (−)- the variable was not carried forward to the multivariate analysis

Stunted growth was associated with lower scores in nonverbal reasoning (β = − 0.16, *p* = 0.030); working memory (β = − 0.20, *p* = 0.027); visual attention (β = − 0.20, *p* = 0.023); syntax (β = − 0.29, *p* = 0.000); word finding (β = 0.30, *p* = 0.001); memory (β = 0.31, *p* = 0.001); EGMA (β =0.24, *p* = 0.000); and EGRA (β = 0.24, *p* = 0.000) (Table 5).

Poor caregiver’s mental health was associated with lower scores in word finding (β = − 0.19, *p* = 0.043), while abnormal delivery was associated with lower scores in syntax (β = − 0.16, *p* = 0.041). Hospital admission was associated with lower scores in perceptual motor functioning (β = − 0.19, *p* = 0.024), while neurological problems were associated with lower scores in pragmatics (β = − 0.21, *p* = 0.038) and word finding (β = − 0.20, *p* = 0.043).

## Discussion

The purpose of this study was to establish the long-term neurocognitive and educational outcomes and their correlates in school-aged survivors of NNJ and HIE.

### Neurocognitive and educational outcomes in neonatal jaundice

We found significant differences in word-finding and memory between the NNJ group and the comparison group, whereby, the comparison group performed better. This result suggests that NNJ potentially accentuates the severity of neurocognitive impairment. Similar findings are reported by Chen et al. [19], who state that survivors of NNJ had significantly more language and speech problems compared to the comparison groups. However, the mechanism by which the heightened bilirubin levels associated with NNJ affects language and speech is not well understood and require further investigation.

Our findings indicate that most of the assessed domains (non-verbal reasoning, planning and problem solving, working memory, visual attention, syntax, pragmatics, mathematical and reading ability) are not impacted. Our study supports findings by Seidman et al. [18], Newman et al. [20], and Chen et al. [19] who did not find any differences in cognitive or intelligence impairment between the school-aged survivors of NNJ and the comparison group. The lack of significant differences in outcomes could be because of the plasticity of the brain that might have compensated for the damaged cells during the first few years of life before the critical period of neurocognitive maturation elapses [50, 51]. Therefore, the survivors of NNJ could have normal development attuned to the environment in which they grew up since the sensory experiences and language stimulation during the first 3 years may determine myelination, synaptogenesis, and neuronal connectivity. Similar effects have been found even in children born with serious brain damage due to very low birth weight [52].

### Correlates of neurocognitive and educational outcomes in neonatal jaundice

Our study identified abnormal pregnancy and febrile seizures as underlying factors associated with poor neurocognitive and educational outcomes in NNJ. To our knowledge, no other studies have linked these factors to neurocognitive and educational outcomes in NNJ. Durkin et al. (2000) conducted an epidemiology study to identify factors associated with developmental outcomes in children and reported that perinatal difficulties such as abnormal pregnancies were associated with intellectual disability among 2- to 9-year-old children in Pakistan [53].

### Neurocognitive and educational outcomes in hypoxic-ischemic encephalopathy

In this study we found that survivors of HIE scored poorer on pragmatics but did not differ in planning, visual attention, working memory, memory, syntax, and mathematical and reading skills in comparison with the unaffected group. However, the survivors of HIE performed better in non-verbal reasoning compared to the unaffected group. Several studies have reported similar results. Thomson et al. [23] reported that survivors of HIE had slightly better intellectual functioning compared to the comparison group. Marlow et al. [15] reported that survivors of moderate HIE had cognitive abilities similar to the comparison group [15], but found poorer memory and executive functions and more profound disability in survivors of HIE, which was not confirmed in our study. However, it should be noted that in the Marlow et al. study these differences were seen only in severe HIE, yet, in our study, we were not able to categorise the severity of HIE due to lack of Apgar scores.

### Correlates of neurocognitive and educational outcomes in hypoxic-ischemic encephalopathy

Our findings suggest that stunted growth, poor caregiver’s mental health, and hospital admission were associated with poor neurocognitive and educational outcomes in survivors of HIE. As per our knowledge, there were no studies that have linked these factors to neurocognitive and educational outcomes in HIE. However, studies with other populations have found that in children under 3 years, anthropometric status had a direct association with psychomotor scores [47]. Similarly, Durkin et al. also identified malnourishment as a risk for intellectual disability in two to nine-year-old children [54]. A study by Mung’ala-Odera (2006) identified hospital admissions as a risk to neurological impairment in the general population of children aged 6 to 9 years [55].

### Limitations of the study

The caregivers of the participants may have suffered recall bias, especially about the medical history of their children at the neonatal stage. Additionally, we could not perform subgroup analysis based on the severity of HIE as there was limited data on the Apgar score of the children with HIE. Furthermore, there is likely survivor bias as most of the survivors with severe outcomes may have died. Over inclusion of participants with mild or less visible outcomes may have made it difficult to detect differences in outcomes between survivors and the healthy comparison group.

## Conclusion

Compared to healthy-born children, school-aged survivors of NNJ and HIE have considerably poorer outcomes in the various domains that may hinder their functioning. Given the strong evidence based on the negative effects of stunted growth, poor caregiver’s mental health, hospital admissions, abnormal pregnancy, and febrile seizures on neurodevelopmental outcomes of at-risk children, our results suggest the need for the implementation of early intervention measures to enhance outcomes among survivors of NNJ and HIE. The development of children with NNJ and HIE need to be monitored after discharge from the hospitals and at subsequent years. Future studies should use a longitudinal design to follow-up participants and investigate the extent to which NNJ and HIE contribute to the neurocognitive and educational outcomes in the presence of the correlates identified in this study as this gives insights into causality and potential interventions required. Also, future studies should incorporate measurements on severity of NNJ and HIE to estimate the impact of severity on outcomes.

## Data Availability

The datasets analysed during the current study are available in the Harvard dataverse, 10.7910/DVN/BB4SKI.

## Abbreviations

CGMR-C: Centre for geographic medicine research - coast
EGMA: Early grade mathematical assessment
EGRA: Early grade reading assessment
FEQ: Family environment questionnaire
HAZ: Height-for-age
KCH: Kilifi county hospital
KHDSS: Kilifi health demographic surveillance system
HIE: Hypoxic-ischemic encephalopathy
KCBMT: Kilifi creek behavioural memory test
KEMRI: Kenya medical research institute
NNJ: Neonatal jaundice
PHQ-9: Patient health questionnaire-9
RCPM: Ravens coloured progressive matrices
SERU: Scientific ethics review unit
TSB: Total serum bilirubin
WAZ: Weight-for-age

## References

[1] English M, Ngama M, Musumba C, Wamola B, Bwika J, Mohammed S (2003). Causes and outcome of young infant admissions to a Kenyan district hospital. Arch Dis Child.

[2] Slusher TM, Zamora TG, Appiah D, Stanke JU, Strand MA, Lee BW (2017). Burden of severe neonatal jaundice: a systematic review and meta-analysis. BMJ Paediatr Open.

[3] Mwaniki MK, Atieno M, Lawn JE, Newton CR (2012). Long-term neurodevelopmental outcomes after intrauterine and neonatal insults: a systematic review. Lancet.

[4] Gordon AL, English M, Tumaini Dzombo J, Karisa M, Newton CR (2005). Neurological and developmental outcome of neonatal jaundice and sepsis in rural Kenya. Tropical Med Int Health.

[5] Olusanya B, Akande A, Emokpae A, Olowe S (2009). Infants with severe neonatal jaundice in Lagos, Nigeria: incidence, correlates and hearing screening outcomes. Tropical Med Int Health.

[6] Wolf M-J, Wolf B, Beunen G, Casaer P (1999). Neurodevelopmental outcome at 1 year in Zimbabwean neonates with extreme hyperbilirubinaemia. Eur J Pediatr.

[7] Finer N, Robertson C, Richards R, Pinnell L, Peters K (1981). Hypoxic-ischemic encephalopathy in term neonates: perinatal factors and outcome. J Pediatr.

[8] Robertson CM, Finer NN (1993). Long-term follow-up of term neonates with perinatal asphyxia. Clin Perinatol.

[9] Barnett A, Mercuri E, Rutherford M, Haataja L, Frisone M, Henderson S (2002). Neurological and perceptual-motor outcome at 5-6 years of age in children with neonatal encephalopathy: relationship with neonatal brain MRI. Neuropediatrics..

[10] de Vries LS, Jongmans MJ (2010). Long-term outcome after neonatal hypoxic-ischaemic encephalopathy. Arch Dis Child Fetal Neonatal Ed.

[11] Hokkanen L, Launes J, Michelsson K (2014). Adult neurobehavioral outcome of hyperbilirubinemia in full term neonates—a 30 year prospective follow-up study. Peer J.

[12] Kuzniewicz M, Newman TB (2009). Interaction of hemolysis and hyperbilirubinemia on neurodevelopmental outcomes in the collaborative perinatal project. Pediatrics..

[13] Mañ, eru C, Serra-Grabulosa JM, Junqué C, Salgado-Pineda P, Bargalló N (2003). Residual hippocampal atrophy in asphyxiated term neonates. J Neuroimaging.

[14] Mañeru C, Junqué C, Botet F, Tallada M, Guardia J (2001). Neuropsychological long-term sequelae of perinatal asphyxia. Brain Inj.

[15] Marlow N, Rose A, Rands C, Draper E (2005). Neuropsychological and educational problems at school age associated with neonatal encephalopathy. Arch Dis Child Fetal Neonatal Ed.

[16] Robertson C, Finer N, Grace M (1989). School performance of survivors of neonatal encephalopathy associated with birth asphyxia at term. J Pediatr.

[17] Robertson CM, Finer NN. Educational readiness of survivors of neonatal encephalopathy associated with birth asphyxia at term. J Dev Behav Pediatr. 1988. 2976068

[18] Seidman DS, Paz I, Stevenson DK, Laor A, Danon YL, Gale R (1991). Neonatal hyperbilirubinemia and physical and cognitive performance at 17 years of age. Pediatrics..

[19] Chen M-H, Su T-P, Chen Y-S, Hsu J-W, Huang K-L, Chang W-H (2014). Is neonatal jaundice associated with autism spectrum disorder, attention deficit hyperactivity disorder, and other psychological development? A nationwide prospective study. Res Autism Spectr Disord.

[20] Newman TB, Klebanoff MA (1993). Neonatal hyperbilirubinemia and long-term outcome: another look at the collaborative perinatal project. Pediatrics..

[21] Amin SB, Prinzing D, Myers G (2009). Hyperbilirubinemia and language delay in premature infants. Pediatrics..

[22] Öğün B, Şerbetçioğlu B, Duman N, Özkan H, Kırkım G (2003). Long-term outcome of neonatal hyperbilirubinaemia: subjective and objective audiological measures. Clin Otolaryngol Allied Sci.

[23] Thomson AJ, Searle M, Russell G (1977). Quality of survival after severe birth asphyxia. Arch Dis Child.

[24] Maimburg RD, Bech BH, Vaeth M, Møller-Madsen B, Olsen J (2010). Neonatal jaundice, autism, and other disorders of psychological development. Pediatrics..

[25] Bronfenbrenner U (2005). Making human beings human: bioecological perspectives on human development: sage.

[26] Scott JAG, Bauni E, Moisi JC, Ojal J, Gatakaa H, Nyundo C (2012). Profile: the Kilifi health and demographic surveillance system (KHDSS). Int J Epidemiol.

[27] Avery GB (2005). Avery’s neonatology: pathophysiology & management of the newborn: Lippincott Williams & wilkins.

[28] Ho NK (1992). Neonatal jaundice in Asia. Baillieres Clin Haematol.

[29] Kramer LI (1969). Advancement of dermal icterus in the jaundiced newborn. Am J Dis Children.

[30] Porter ML, Dennis BL (2002). Hyperbilirubinemia in the term newborn. Am Fam Physician.

[31] Rijken M, Rijken J, Papageorghiou A, Kennedy S, Visser G, Nosten F (2011). Malaria in pregnancy: the difficulties in measuring birthweight. BJOG Int J Obstet Gynaecol.

[32] Taylor R, Denison F, Beyai S, Owens S (2010). The external Ballard examination does not accurately assess the gestational age of infants born at home in a rural community of the Gambia. Ann Trop Paediatr.

[33] Bhutani V, Johnson L (2009). Kernicterus in the 21st century: frequently asked questions. J Perinatol.

[34] Smitherman H, Stark AR, Bhutan VK (2006). Early recognition of neonatal hyperbilirubinemia and its emergent management. Seminars in fetal and neonatal medicine.

[35] Varughese PM (2019). Kramer’s scale or transcutaneous bilirubinometry: the ideal choice of a pediatrician? Can we trust our eyes?. Int J Contemp Pediatr.

[36] World Health Organization (WHO) (2005). Pocket book of hospital care for children: guidelines for the management of common illnesses with limited resources: World Health Organization.

[37] Raven JC (1983). Manual for Raven’s progressive matrices and vocabulary scales. Standard progressive matrices.

[38] Kitsao-Wekulo PK, Holding PA, Taylor HG, Abubakar A, Connolly K (2013). Neuropsychological testing in a rural African school-age population: evaluating contributions to variability in test performance. Assessment..

[39] Shallice T (1982). Specific impairments of planning. Philos Trans R Soc Lond B.

[40] Holding PA, Taylor HG, Kazungu SD, Mkala T, Gona J, Mwamuye B (2004). Assessing cognitive outcomes in a rural African population: development of a neuropsychological battery in Kilifi District, Kenya. J Int Neuropsychol Soc.

[41] Carter J, Murira G, Ross A, Mung'ala-Odera V, Newton C (2003). Speech and language sequelae of severe malaria in Kenyan children. Brain Inj.

[42] Kihara M, Carter JA, Holding PA, Vargha-Khadem F, Scott RC, Idro R (2009). Impaired everyday memory associated with encephalopathy of severe malaria: the role of seizures and hippocampal damage. Malar J.

[43] Piper B, Zuilkowski SS, Mugenda A (2014). Improving reading outcomes in Kenya: first-year effects of the PRIMR initiative. Int J Educ Dev.

[44] World Health Organization (WHO) (2007). WHO Anthro for personal computers manual. Software for assessing growth and development of the World’s children.

[45] Spitzer RL, Kroenke K, Williams JB, Group PHQPCS (1999). Validation and utility of a self-report version of PRIME-MD: the PHQ primary care study. JAMA..

[46] Vostanis P, Nicholls J (1995). The family environment scale: comparison with the construct of expressed emotion. J Fam Ther.

[47] Abubakar A, Van de Vijver F, Van Baar A, Mbonani L, Kalu R, Newton C (2008). Socioeconomic status, anthropometric status, and psychomotor development of Kenyan children from resource-limited settings: a path-analytic study. Early Hum Dev.

[48] World Health Organization (WHO) (2009). WHO AnthroPlus for personal computers: software for assessing growth of the world’s children and adolescents.

[49] Kariuki SM, Abubakar A, Holding PA, Mung'ala-Odera V, Chengo E, Kihara M (2012). Behavioral problems in children with epilepsy in rural Kenya. Epilepsy Behav.

[50] Kolb B, Harker A, Gibb R (2017). Principles of plasticity in the developing brain. Dev Med Child Neurol.

[51] Mundkur N (2005). Neuroplasticity in children. Indian J Pediatr.

[52] Weisglas-Kuperus N, Koot HM, Baerts W, Fetter WP, Sauer PJ (1993). Behaviour problems of very lowbirthweight children. Dev Med Child Neurol.

[53] Durkin M, Khan N, Davidson L, Huq S, Munir S, Rasul E (2000). Prenatal and postnatal risk factors for mental retardation among children in Bangladesh. Am J Epidemiol.

[54] Durkin MS, Hasan Z, Hasan K (1998). Prevalence and correlates of mental retardation among children in Karachi, Pakistan. Am J Epidemiol.

[55] Mung’ala-Odera V, Meehan R, Njuguna P, Mturi N, Alcock KJ, Newton C (2006). Prevalence and risk factors of neurological disability and impairment in children living in rural Kenya. Int J Epidemiol.

